# Loss of hormone receptor expression in breast tissue following pre‐surgical therapy with an SERD

**DOI:** 10.1111/his.70028

**Published:** 2025-10-31

**Authors:** Francesco de Napoli, Bethania Fernandes, Silvia Uccella, Margherita D'Arezzo, Martina Iuzzolino, Luca Di Tommaso

**Affiliations:** ^1^ Unit of Pathology IRCCS Humanitas Research Hospital Milan Italy; ^2^ Department of Biomedical Sciences Humanitas University Milan Italy

**Keywords:** breast, hormone, neoadjuvant, receptors, treatment

**Abbreviations**
PR: progesterone receptors
SERD: Selective Oestrogen Receptor Degraders

Oestrogen receptors (ER) play a central role to breast carcinogenesis, making anti‐oestrogen therapies a cornerstone in treatment for luminal‐type tumours.^1^ Over the last years, Selective Oestrogen Receptor Degraders (SERDs) are being developed, but their effects on both neoplastic and non‐neoplastic breast tissue remain poorly defined. Here, we present three cases in which SERD treatment was associated with unexpected histological changes in normal mammary tissue.

Three female patients, aged between 39 and 48 years, underwent core needle biopsy for a clinical diagnosis of breast carcinoma. All tumours were diagnosed as No Special Type G2 and showed strong and diffuse positivity for ER (>90%) and progesterone receptors (PR, >90%), with high proliferation indices (Ki67, 10%–40%), while HER2 status was negative.

One month after the biopsy, all patients underwent quadrantectomy. Surgical specimens were examined intraoperatively for macroscopic evaluation and subsequently processed according to standard fixation protocols. At histology, initial immunohistochemical assessment revealed an apparent loss of ER and PR expression, both within the tumour and in the adjacent non‐neoplastic mammary tissue (Figure 1). Initially, fixation artefacts were considered; however, after discussion with the treating clinicians, we learned that all patients had received SERD therapy for about 4 weeks between biopsy and surgery.

**Figure 1:**
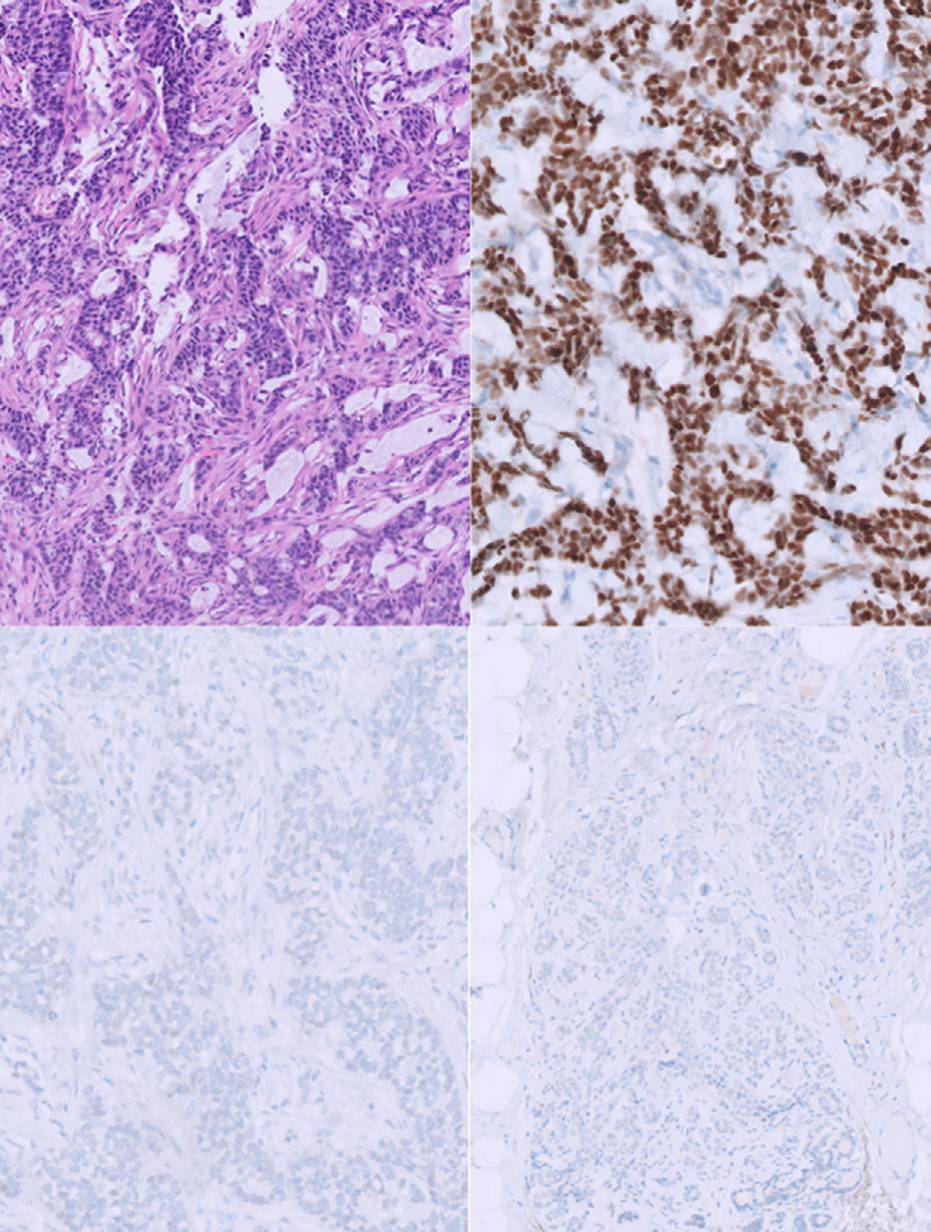
Case 1. Top row, biopsy specimen: on the left, tumour morphology on EE staining; on the right, ER expression in neoplastic tissue. Bottom row, surgical specimen: on the left, ER expression in neoplastic tissue; on the right, ER expression in non‐neoplastic tissue.

Once realized this important information, a closer inspection at higher magnification revealed faint nuclear ER staining in the benign parenchyma in all three cases (Figure 1). This internal control prompted a re‐evaluation of the neoplastic tissue, revealing very low‐intensity expression of ER (60%–90%) and PR (5%–90%), while Ki67 had markedly decreased (2%–10%). In two cases, HER2 status showed an increase in immunohistochemical scoring from baseline (from 0–1 to 2+) (Figure 2), although FISH testing showed no gene amplification.

**Figure 2:**
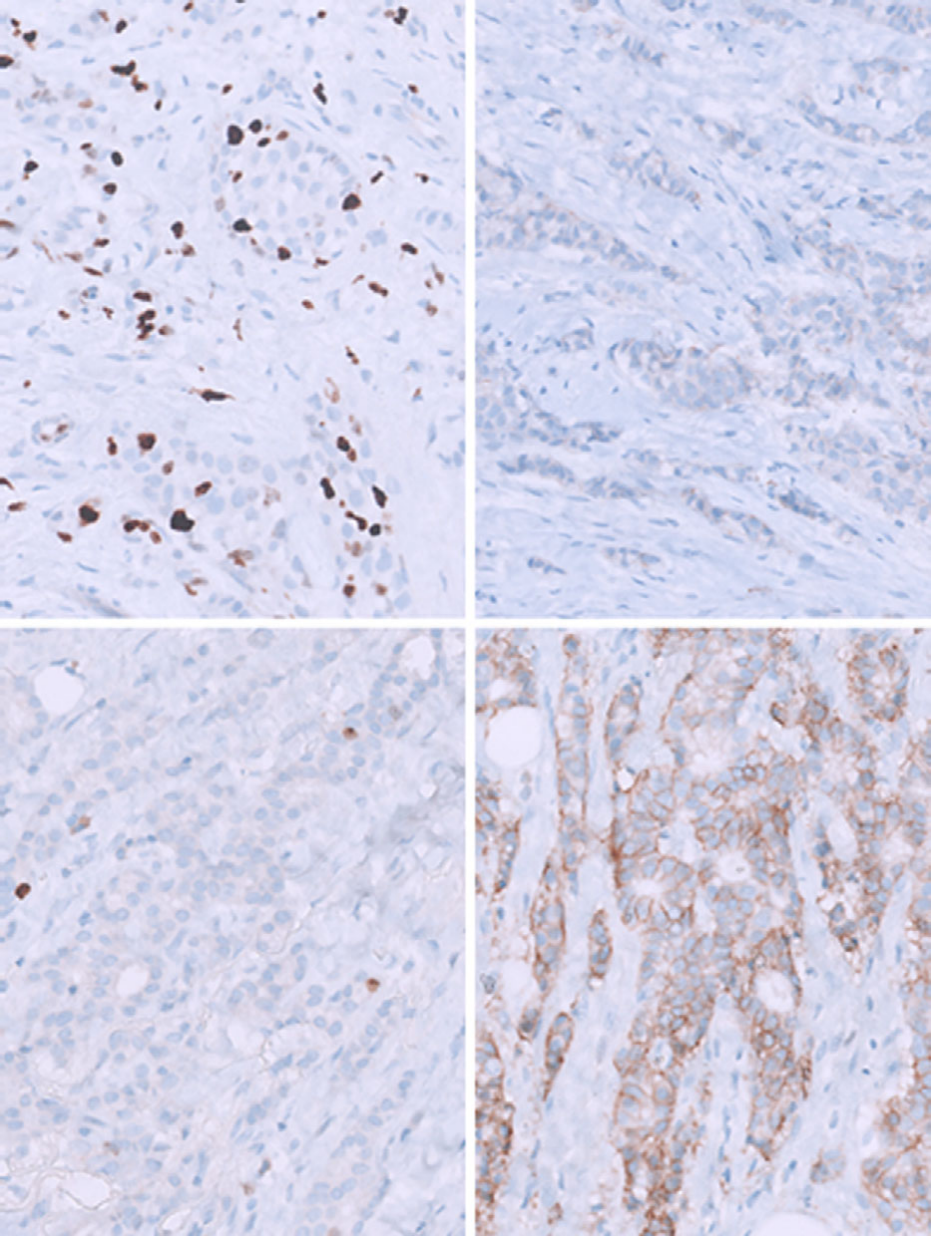
Case 1. Comparison of Ki67 (on the left) and HER2 expression (on the right) in neoplastic tissue. Top row: biopsy specimen. Bottom row: surgical specimen.

The three cases described in this report highlight a known but potentially risky phenomenon for pathologists. SERDs are well known to induce ER degradation in cancer cells, and it is reasonable to assume that they can also diminish ER and PR expression in benign glands. This phenomenon is rooted in their molecular mechanism of action. SERDs bind to the ER ligand‐binding domain and induce a conformational change that disrupts the receptor's stability. The altered conformation promotes ubiquitin ligases recruitment, resulting in accelerated degradation of ER via the proteasome.^1^ If treatment history is not taken into account, an unaware observer might reclassify the cancer as a hormone receptor‐negative phenotype, and this could dramatically alter postoperative management or raise questions about whether the initial biopsy was mistaken or whether a second primary tumour was present.

Moreover, these three cases revealed a further unexpected finding: the shift in HER2 immunohistochemical score. Indeed, prolonged ER inibition sometimes leads to upregulation of alternative growth pathways in a subset of cells, as a mechanism of resistance. For instance, long‐term fulvestrant treatment was associated with modest increases in HER2 expression in ~29% of tumours.^2^ Loss of ER input may relieve feedback inhibition on growth factor receptors or induce cells to rely more on HER2/EGFR signalling. These resistance mechanisms remind us that the pronounced initial suppression of ER/PR/Ki‐67 by SERDs might not be permanent if the tumour eventually activates escape pathways.^3^

It is worth noting that some of the clinical trials conducted for SERD approval adopted the change in tumour Ki67 as a primary endpoint. A Ki67 value below 2.7% is considered indicative of complete cell cycle arrest according to ESMO guidelines and is regarded as a strong biomarker of endocrine therapy response, correlating with excellent prognosis.^4^

In conclusion, the presented cases illustrate that a short course of a novel SERD can almost abolish ER and PR from a luminal breast carcinoma and halt its proliferation, also affecting normal breast tissue. This finding, while exciting from a treatment perspective, could easily be misinterpreted without the proper context. Moreover, the indirect effect on HER2 expression warrants further investigation in order to clarify its biological significance and potential clinical implications.

## Author contributions

All authors contributed to the study conception and design. Material preparation, data collection and analysis were performed by Dr. Francesco de Napoli and Prof. Luca Di Tommaso. The first draft of the manuscript was written by Francesco de Napoli and all authors commented on previous versions of the manuscript. All authors read and approved the final manuscript.

## Funding information

No funding was received to assist with the preparation of this manuscript.

## Conflict of interests

The authors have no competing interests to declare that are relevant to the content of this article.

## Data Availability

The data that support the findings of this study are available from the corresponding author upon reasonable request.
